# Perceived Gaze Direction Differentially Affects Discrimination of Facial Emotion, Attention, and Gender – An ERP Study

**DOI:** 10.3389/fnins.2019.00517

**Published:** 2019-05-24

**Authors:** Sarah D. McCrackin, Roxane J. Itier

**Affiliations:** Department of Psychology, University of Waterloo, Waterloo, ON, Canada

**Keywords:** gaze direction, attention discrimination, emotion discrimination, gender discrimination, face perception, ERP

## Abstract

The perception of eye-gaze is thought to be a key component of our everyday social interactions. While the neural correlates of direct and averted gaze processing have been investigated, there is little consensus about how these gaze directions may be processed differently as a function of the task being performed. In a within-subject design, we examined how perception of direct and averted gaze affected performance on tasks requiring participants to use directly available facial cues to infer the individuals’ emotional state (emotion discrimination), direction of attention (attention discrimination) and gender (gender discrimination). Neural activity was recorded throughout the three tasks using EEG, and ERPs time-locked to face onset were analyzed. Participants were most accurate at discriminating emotions with direct gaze faces, but most accurate at discriminating attention with averted gaze faces, while gender discrimination was not affected by gaze direction. At the neural level, direct and averted gaze elicited different patterns of activation depending on the task over frontal sites, from approximately 220–290 ms. More positive amplitudes were seen for direct than averted gaze in the emotion discrimination task. In contrast, more positive amplitudes were seen for averted gaze than for direct gaze in the gender discrimination task. These findings are among the first direct evidence that perceived gaze direction modulates neural activity differently depending on task demands, and that at the behavioral level, specific gaze directions functionally overlap with emotion and attention discrimination, precursors to more elaborated theory of mind processes.

## Introduction

Eye-gaze has long been considered one of the most important cues during social interactions and seems central to social cognition (Kleinke, 1986; Emery, 2000; George and Conty, 2008; Itier and Batty, 2009 for reviews). Perceiving eye-gaze is thought to be a key component of theory of mind, our ability to infer what others are feeling and thinking (Baron-Cohen and Cross, 1992). This “language of the eyes” informs how we respond and interact with those around us (Baron-Cohen et al., 1997). The importance of eye-gaze is especially evident in populations who display behavioral avoidance of the eye region as well as social impairment, including Autism Spectrum Disorder (Pelphrey et al., 2002; Senju and Johnson, 2009; Madipakkam et al., 2017) and Social Anxiety Disorder (Schneier et al., 2011).

There is support for the idea that key differences exist between the processing of direct and averted gaze. Direct gaze has been heavily implicated in emotion processing (see Hamilton, 2016 for a review), as it is associated with increased ventral striatum activation (Kampe et al., 2001; Strick et al., 2008; see Cardinal et al., 2002, for a review of the ventral striatum’s implication in emotion processing). It is behaviorally more arousing than averted gaze (Nichols and Champness, 1971; Conty et al., 2010; McCrackin and Itier, 2018c) and it has been shown that participants are better at reporting their own emotional state after seeing direct gaze faces than averted gaze faces (Baltazar et al., 2014). While both gaze directions inform an observer about the gazer’s attentional state, seeing averted gaze informs an observer about the object or environment that the gazer is looking at (George and Conty, 2008; Itier and Batty, 2009). Perceived averted gaze also spontaneously orients the perceiver’s attention toward the gazed-at location (Friesen and Kingstone, 1998; Driver et al., 1999; see Frischen et al., 2007 for a review) and this gaze cueing is even faster if the gazer is smiling or fearful, which likely helps the perceiver attend faster to environmental threats or rewards (e.g., McCrackin and Itier, 2018a,b). In contrast, direct gaze is self-referential, indicating that the observer is the focus of attention (George and Conty, 2008; Itier and Batty, 2009; Conty et al., 2016), and direct gaze has been shown to produce similar brain activation as hearing one’s name being called (Kampe et al., 2003).

Accumulating evidence from neuroimaging studies suggests that eye-gaze is processed by a complex brain network whose nodes include the superior temporal sulcus, the amygdala, the medial prefrontal cortex, the orbitofrontal cortex, and parietal regions such as the intraparietal sulcus (for reviews, see Grosbras et al., 2005; George and Conty, 2008; Itier and Batty, 2009; Nummenmaa and Calder, 2009). However, inconsistencies in brain activity linked to the processing of direct and averted gaze have been noted. For instance, some have found increased superior temporal sulcus activation for direct gaze relative to averted gaze (Calder et al., 2002; Wicker et al., 2003; Pelphrey et al., 2004) while others have found the opposite (Hoffman and Haxby, 2000), or no difference in activation between the two gaze types (Wicker et al., 1998; Pageler et al., 2003). Similarly, the orbitofrontal cortex has been reported to show increased activation for direct than averted gaze (Wicker et al., 2003), or no gaze difference (Wicker et al., 1998), and the amygdala has been found to be more active for direct than averted gaze (Kawashima et al., 1999; George et al., 2001), more active for averted than direct gaze (Hooker et al., 2003; Wicker et al., 2003), or not active at all (Pageler et al., 2003).

Most importantly for the present study, similar inconsistencies have been reported in the Event Related Potential (ERP) literature, which attempts to track the time-course of gaze processing. A large proportion of studies have focused on the N170, a face-sensitive ERP component that occurs approximately 130–200 ms post face presentation over occipitotemporal sites, and is thought to reflect the structural encoding of the face (Bentin et al., 1996; George et al., 1996; Eimer, 2000). Some have found this component to be larger for averted gaze faces or averted gaze shifts (Puce et al., 2000; Watanabe et al., 2002; Itier et al., 2007; Latinus et al., 2015; Rossi et al., 2015), while others have found it to be larger for direct gaze static faces or direct gaze shifts (Watanabe et al., 2006; Conty et al., 2007; Pönkänen et al., 2010; Burra et al., 2017), yet others have found no N170 gaze effect at all (Taylor et al., 2001; Schweinberger et al., 2007; Brefczynski-Lewis et al., 2011). Gaze modulations have also been reported before the N170, around 100–140 ms with both greater amplitudes for direct than averted gaze (e.g., Burra et al., 2018) and greater amplitudes for averted gaze than direct gaze (Schmitz et al., 2012). Finally, gaze effects have been reported after the N170, around 250–350 ms (adaptation study looking at left/right gaze directions, Schweinberger et al., 2007) or even 300–600 ms with greater direct gaze than averted gaze amplitudes (Conty et al., 2007; Itier et al., 2007; Burra et al., 2018) or vice versa (Carrick et al., 2007).

One likely contributor to these inconsistencies is the type of experimental paradigm used. Common tasks given to participants while they are shown direct and averted gaze images include oddball tasks (i.e., responding to an infrequent stimulus presented among frequent other stimuli; e.g., Brefczynski-Lewis et al., 2011; Rossi et al., 2015; Burra et al., 2018) and passive viewing tasks (Puce et al., 2000; George et al., 2001; Taylor et al., 2001; Watanabe et al., 2002, 2006; Pönkänen et al., 2010), as well as tasks requiring the discrimination of gender (Burra et al., 2018), gaze direction (Hoffman and Haxby, 2000; Hooker et al., 2003; Conty et al., 2007; Itier et al., 2007; Schweinberger et al., 2007; Latinus et al., 2015), emotional expression (Akechi et al., 2010), identity (Hoffman and Haxby, 2000), or head orientation (Itier et al., 2007). These different task demands likely contribute to the reported inconsistencies regarding which brain areas are more involved for which gaze direction, and the time course of this gaze processing difference. While both the ERP and the neuroimaging literatures have begun to explore how eye-gaze processing differs based on what participants are asked to do (Hoffman and Haxby, 2000; Hooker et al., 2003; Carrick et al., 2007; Latinus et al., 2015; Burra et al., 2018), few studies have employed direct task comparisons within the same participants. Within-subject designs are, however, more powerful statistically than between-subject designs and are necessary to draw conclusions regarding possible task effects on the neural processing of direct versus averted gaze.

As far as we know, the limited number of within-subject ERP studies that have directly compared tasks, have focused on the processing of facial expressions of emotion, using Gender Discrimination (GD) and Emotion Discrimination (ED) judgments. The stimuli used were eye-region stimuli (Sabbagh et al., 2004) or faces (Wronka and Walentowska, 2011; Rellecke et al., 2012; Itier and Neath-Tavares, 2017), but always with direct gaze. One exception includes the comparison of an ED task to judgments of looking direction and of object choice based on averted gaze faces only (Cao et al., 2012). These studies suggest that ED and GD tasks differentiate mainly after the N170 component. While Rellecke et al. (2012) and Wronka and Walentowska (2011) found no ERP difference between the two tasks, Sabbagh et al. (2004) found that the ED task resulted in more negative ERPs than the GD task over inferior frontal and anterior temporal sites from 270 to 400 ms, which source localization suggested was driven by orbitofrontal and medial temporal activation. The ED task also resulted in more positive ERPs than the GD task from 300 to 500 ms over posterior central and parietal sites (Sabbagh et al., 2004), a similar finding to Itier and Neath-Tavares’s (2017) report of more positive ERPs elicited by the GD task than the ED task over posterior sites from 200 to 350 ms (the latest tested time-window).

To the best of our knowledge, no current ERP study has directly investigated task effects on the processing of direct versus averted gaze faces in a within-subject design. The present study begins to fill this gap by examining the time-course of direct and averted gaze perception within three different discrimination tasks that have been commonly used in the gaze processing literature. Using the exact same stimuli for each task, i.e., male and female faces expressing anger or joy and with direct or averted eye-gaze, participants indicated whether the face expressed anger or joy (ED task), whether the face was male or female (GD task) and whether the face was attending to them or away from them (Attention Discrimination – AD task). Importantly, explicit processing of gaze direction was required by the AD task while gaze was irrelevant to the GD and ED tasks. ERPs time-locked to the presentation of the face stimuli were used to track the time-course of when gaze and task processing were occurring and interacting. If direct and averted gaze differentially impacted these three cognitive processes, we expected to see dissociations at the neural level, in spatial location (different electrodes) and/or in the time course of the interaction, as well as at the behavioral level.

Given the mixed findings reported on the N170 component as reviewed earlier, we analyzed a cluster of occipitotemporal electrodes during the time window encompassing this component (130–220 ms). However, the findings from the gaze and ERP literature on different tasks suggested that we might pick up a gaze and task interaction over frontal sites between 200 and 400 ms post-stimulus, after both gaze (e.g., Puce et al., 2000; Watanabe et al., 2002; Itier et al., 2007; Latinus et al., 2015; Rossi et al., 2015) and ED and GD task differences (Sabbagh et al., 2004) are processed. As gaze effects are traditionally picked up over parieto-occipital sites (Itier and Batty, 2009), and posterior central and parietal sites have been shown to discriminate between ED and GD tasks from 200 to 500 ms (Sabbagh et al., 2004; Cao et al., 2012; Itier and Neath-Tavares, 2017), we also hypothesized that we may find an interaction between gaze and task over posterior sites from 200 to 500 ms.

It has to be highlighted that the ERP field is witnessing a transition toward more robust data analyses. As Luck and Gaspelin (2017) recently demonstrated, examining the ERP waveforms (typically the group grand-average) before deciding which electrodes and time-windows to analyze, can massively inflate type I errors and lead to reporting false effects. Similarly, although using *a priori* hypotheses to select electrodes and time-windows provides resistance to type I errors, this approach can prevent the discovery of real effects at untested time-points. As most of the ERP literature on gaze processing employed both of these classic approaches, it is possible that a lot of the inconsistencies reported in the time course of the effect were also due, in addition to the various task demands, to the way the analyses were performed. While there is no perfect solution, the mass univariate approach shows promise in its capacity to reduce both types of error (Groppe et al., 2011; Pernet et al., 2011, 2015; Luck and Gaspelin, 2017; Fields and Kuperberg, 2018). With this approach, hypothesis testing can first be performed on a subset of *a priori* electrodes and time-points with a multiple comparison correction applied to control for type I errors (Groppe et al., 2011). Then, an exploratory analysis can be performed by testing each electrode at every time-point to enable the discovery of unpredicted effects, with the caveat that this type of analysis can have weak power because of the number of comparisons corrected for. Accordingly, we used the freely available Factorial Mass Univariate Toolbox (FMUT) extension (Fields, 2017) for the Mass Univariate Toolbox (MUT; Groppe et al., 2011) to perform a mass-univariate analysis in the present study. We first performed our hypothesis testing by running a mass univariate analysis on occipitotemporal sites from 130 to 200 ms to capture the N170, at frontal sites from 200 to 400 ms, and on parieto-occipital sites from 300 to 500 ms. Then we performed an exploratory analysis over all electrodes and time-points.

## Materials and Methods

### Participants

Thirty-six undergraduate students from the University of Waterloo (UW) participated in the study and received course credit upon completion. All were 18–29 years old, had normal or corrected-to-normal vision and had lived in Canada or the United States for the past 5 years or more. They reported no history of neurological or psychiatric illness and no drug use (psychiatric or otherwise). All participants rated themselves at least a 7 out of 10 on Likert-type scales when describing their ability to recognize people and emotional expressions (from 0-extremely poor to 10-extremely good). In total, ten participants were excluded before analysis due to technical issues during recording (*N* = 2), problems with eye-tracking calibration (*N* = 2), poor response accuracy (i.e., less than 80%; *N* = 2), or EEG data that had less than 50 trials per condition after cleaning (*N* = 4). This left a final sample of 26 participants (17 females, 9 males; *mean age* = 19.67, *SD* = 1.69) for analysis. The study received ethics clearance from the UW Research Ethics Board and all participants gave written informed consent in accordance with the Declaration of Helsinki.

### Face Stimuli

**FIGURE 1:**
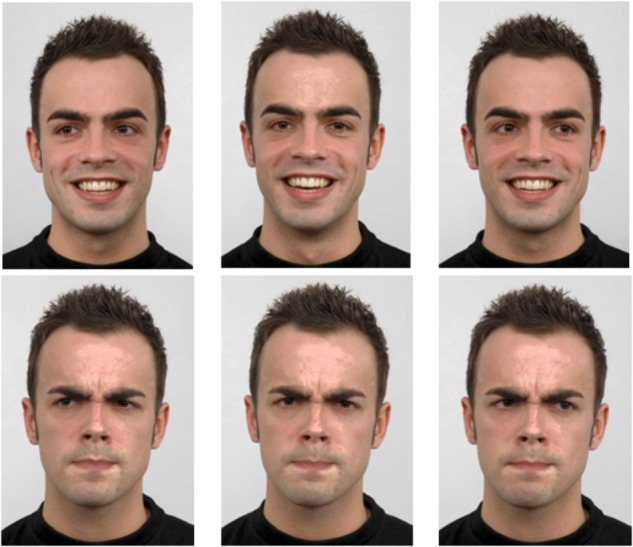
Sample images of one individual with happy and angry expressions displaying direct, averted left and averted right gaze (human image obtained from “Radboud Face dataset,” used with permission – http://www.socsci.ru.nl:8180/RaFD2/RaFD?p=faq).

Five male and five female Caucasian identities were selected from the Radboud database (Langner et al., 2010).^1^ Each individual displayed an angry expression and a happy expression with direct gaze, averted left gaze and averted right gaze (Figure 1). All gaze deviations were of equal magnitude. The images were cropped with the GNU Image Manipulation Program (GIMP 2.8) so that only the individual’s upper shoulders, head and neck were visible. All images were then mirrored to control for any asymmetry between the left and right image halves by creating a second set of images (e.g., an angry averted right image mirrored became a new angry averted left image). Images were equated on mean pixel intensity (*M* = 0.56, *SD* = 0.0003) and root mean square (RMS) contrast (*M* = 0.48, SD = 0.0002) with the SHINE package (Willenbockel et al., 2010). Custom MATLAB scripts were then used to add the color information back into each image for added realism.

### Experimental Design

Participants first provided informed consent, and then filled out a demographic questionnaire. They were fitted with an EEG cap and led to a sound-attenuated faraday cage with dim lighting for the experiment, which was presented on a CRT monitor with a refresh rate of 85 Hz and a resolution of 1280 × 960. A chinrest helped participants keep their heads still at a distance of 65 cm away from the monitor. Participants’ dominant eyes were determined using the Miles test (Miles, 1930) and then tracked at a 1000 Hz sampling rate with an Eyelink 1000 eye-tracker, which was recalibrated whenever necessary.

Participants were told that they would see pictures of individuals and complete three tasks, and that a prompt at the beginning of each trial would let them know which task to perform for that trial. The first task required indicating what emotional state the person was in (Emotion Discrimination Task, hereafter ED task; prompted by the words “Happy/Angry”). The second task required indicating whether the person was directing their attention at them (the participant) or away from them (Attention Discrimination task, hereafter AD task; prompted by “At Me/Away” words). The third task required indicating whether the person was a male or female (Gender Discrimination task, hereafter GD task; prompted by “Male/Female” words). Participants were asked to indicate their answer when prompted using the left and right arrow keys.

**FIGURE 2:**
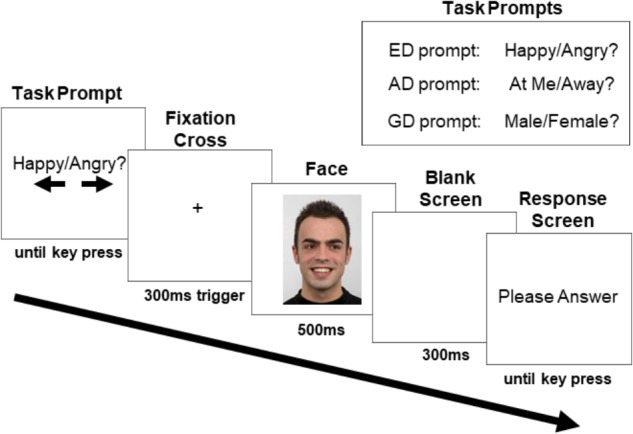
Sample trial progression with an averted gaze trial in the ED task (human image obtained from “Radboud Face dataset,” used with permission (http://www.socsci.ru.nl:8180/RaFD2/RaFD?p=faq). ERPs were recorded to the onset of the face stimulus. The three task prompts are shown in the top right corner.

Figure 2 depicts a typical trial progression. At the trial start, the task prompt appeared, notifying the participant of the task and visually reminding them (with arrows) which answers corresponded to the left and right arrow keys. Task type was randomized and there were an equal number of trials for each task presented in each block. The response mapping for the arrow keys was counterbalanced between participants (i.e., half pressed the right arrow key for “angry,” and half pressed the left arrow key; half pressed the right arrow key for direct gaze and half pressed the left arrow key; half pressed the right arrow key for male and half pressed the left arrow key). Participants were instructed to press the space bar when they had read the prompt, and this key press triggered the appearance of a white screen with a fixation cross (18.43° down on the horizontal midline). Participants were asked to fixate the cross for a minimum of 300 ms within a 1.92° × 1.92° margin to advance the trial to the face screen. This ensured that participants were fixated between the nasion and the nose when the face appeared. If ten seconds elapsed without this requirement being met, a drift correction occurred, canceling the trial. If the requirement was met, the trial advanced by presenting the face image (subtending 10.64° horizontally and 15.08° vertically) on a white background for 500 ms. There were an equal number of direct and averted gaze faces, with half of the averted gaze trials consisting of faces looking to the left and half to the right (all averted gaze trials were grouped together for analysis). Face identity was randomized, and each was presented an equal number of times within each block and within each condition. The face was followed by a 300 ms blank screen after which participants were prompted to indicate their answer by pressing the left or right arrow key. This procedure ensured that the neural activity until 800 ms post face onset would not be contaminated by motor preparation and motor artifacts. However, in doing so, the response times collected were not clearly interpretable and are not further discussed.

SR Research’s Experiment Builder 1.10.1385 was used to program and run the experiment. Participants completed a minimum of four practice trials to ensure they were comfortable with the tasks before starting the study blocks. In total, there were 8 blocks of 96 trials each. There were six within-subject conditions, corresponding to the face’s gaze direction (direct or averted) in each of the three tasks performed (ED, AD, and GD), with facial expression trials collapsed. Thus, across the experiment, there were a total of 128 trials per each of the 6 conditions.

### Electroencephalography Recording

EEG data were recorded with the Active-two Biosemi EEG system at a sampling rate of 512 Hz, time-locked to the presentation of the face stimulus. Electrode offset was kept within a ±20 mV range. There were 66 electrodes on the custom-made caps under the 10/20 system, the 64 classic locations plus PO9 and PO10 electrodes added for increased posterior coverage. In addition, one electrode was placed over each mastoid, infra-orbital ridge, and the outer canthus of each eye, for a total of 72 recording electrodes. A Common Mode Sense (CMS) active-electrode and a Driven Right Leg (DRL) passive-electrode were used as the ground.^2^

### Data Preprocessing and Cleaning

To ensure that participants read the task prompt on each trial, we used the eye-tracking data to exclude trials where participants did not fixate at least twice on the prompt screen within a rectangular region of interest (ROI) spanning the text (subtending 32.71° horizontally and 3.72° vertically, positioned 17.43° down and centered horizontally). This resulted in excluding an average of only 0.81 trials per participant (*SD* = 1.04). We also excluded trials in which participants did not fixate the spot encompassing the eyes, and nasion (a circular 5.50° ROI) that was cued by the fixation cross for at least the first 250 ms of face presentation. As the N170, the earliest face sensitive ERP component, can be modulated by what part of the face is fixated (de Lissa et al., 2014; Nemrodov et al., 2014; Neath and Itier, 2015; Neath-Tavares and Itier, 2016; Itier and Preston, 2018; Parkington and Itier, 2018), this step ensured that fixation location would not play a role in any N170 modulation and that participants were encoding the gaze direction for each face. This resulted in excluding an average of 3.23 trials per participant (*SD* = 4.98). Next, trials with incorrect responses were removed (an average of 4.72 trials/participant, *SD* = 2.09).

EEG data were processed using the EEGLab (version 13.6.5b; Delorme and Makeig, 2004) and ERPLab (version 5.1.1.0^3^) toolboxes in MATLAB 2014b. An average reference was computed offline and data were band-pass filtered (0.01–30 Hz) and then cleaned. Trials were epoched from a -100 ms baseline (before the face) to 800 ms post-face. First, trials were removed if they exceed ±70 μV on any non-frontal and non-ocular channels (i.e., excluding: Fp1, Fpz, Fp2, AF3, AFz, AF4, AF8, AF7, IO1, IO2, LO1, and LO2). Any of these channels that were consistently noisy were removed for later interpolation. Then, data were visually inspected for eye-blinks and saccades. For cases where there were few eye artifacts, the data were manually cleaned, and any removed electrodes were added back in and interpolated with EEGlab’s spherical splines tool. For cases where there were many eye-artifacts, Independent Component Analysis (ICA; using the EEGLab “runica” function) was used to remove saccades and eye-blinks before adding back and interpolating electrodes. Remaining noisy trials were then manually removed when necessary. An average of 97.29 trials/condition (*SD* = 22.34) were included in the final ERP waveforms.^4^

### Data Analysis

#### Behavioral Data Analysis

Correct answers for each condition were those in which the participant pressed the arrow key corresponding to the correct gender (GD task), emotional expression (ED task) or gaze direction (AD task). An ANOVA with the within-subjects factors of gaze direction (2; direct gaze, averted gaze) and task (3; GD, ED, AD) was run on participants’ average accuracy using SPSS 25. Greenhouse-Geisser corrected degrees of freedom were reported when Mauchly’s Test of sphericity was significant. The follow up *t*-tests for the gaze and task interactions were planned based on the theoretical motivation behind this paper. However, for transparency, the raw *p*-values for all follow-up paired *t*-tests are reported, such that those with *p* < 0.05 would be considered significant with Fischer’s LSD test, and those with *p* < 0.016 would be considered significant after Bonferroni-correction (0.05/3 comparisons).

#### EEG Data Analysis

EEG data were analyzed using the Factorial Mass Univariate Toolbox (FMUT) extension (Fields, 2017) for the Mass Univariate Toolbox (MUT; Groppe et al., 2011). FMUT uses robust statistics to test each time-point included in the time-window of interest for the selected electrodes, and then control for the familywise error rate. One ANOVA with the within-subjects factors of gaze direction (2; direct gaze, averted gaze) and task (3; GD, ED and AD) was run over (i) a posterior cluster (P9, P10, PO9. PO10, P7, P8) between 130 and 200 ms encompassing the N170 component, (ii) a frontal electrode cluster (Fp1, Fp2, Fpz, AF3, AF4, AFz, F4, F3, F1, F2, Fz) from 200 to 400 ms, and (iii) parieto-occipital electrodes (Pz, POz, PO4, PO3, P1, P2, Oz, O1, O2) from 200 to 500 ms. The ANOVAs were corrected for multiple comparisons with the Permutation Based Cluster Mass technique (Maris and Oostenveld, 2007; Groppe et al., 2011). With this technique, data points that are spatially and temporally adjacent and that exceed the threshold for inclusion are considered a cluster. All *F*-values in the cluster are then summed, and compared to a null distribution for cluster mass significance estimated with permutations. We used the recommended number of 100,000 permutations and alpha of 0.05, such that clusters exceeding the 1 – α percentile of the resulting distribution were considered significant. As discussed by Groppe et al. (2011) and Maris and Oostenveld (2007), true ERP effects are more likely than noise to occur across multiple adjacent electrodes and time-points, and thus ERP effects will typically stand out more clearly from noise using cluster-based statistics.

Based on the gaze direction by task interaction that we observed in the omnibus ANOVA at frontal sites during 200–400 ms, three follow-up ANOVAs were performed with FMUT to compare the activations associated with direct and averted gaze in each of the three tasks (the use of ANOVAs instead of *t*-tests as follow-up tests is recommended for the Permutation Based Cluster Mass technique; Fields, 2019). We performed these follow up ANOVAs over the frontal sites and time-points (220–290 ms) that were significant in the omnibus ANOVA with an alpha level set to 0.016 to correct for the three comparisons. As in the original ANOVA, 100,000 permutations were calculated.

Finally, we performed an exploratory analysis on all electrodes and relevant time-points (50–800 ms) post-face to allow for the discovery of unpredicted effects, again with 100,000 permutations and an alpha of 0.05. Based on the main effect of task that we observed in this analysis, we performed three follow-up task comparisons over the significant time-points (400–800 ms) and electrodes in the omnibus ANOVA with a corrected alpha level of 0.016.

## Results

The datasets analyzed in the present study are available in the Open Science Framework Repository^5^.

### Participant Accuracy

**FIGURE 3:**
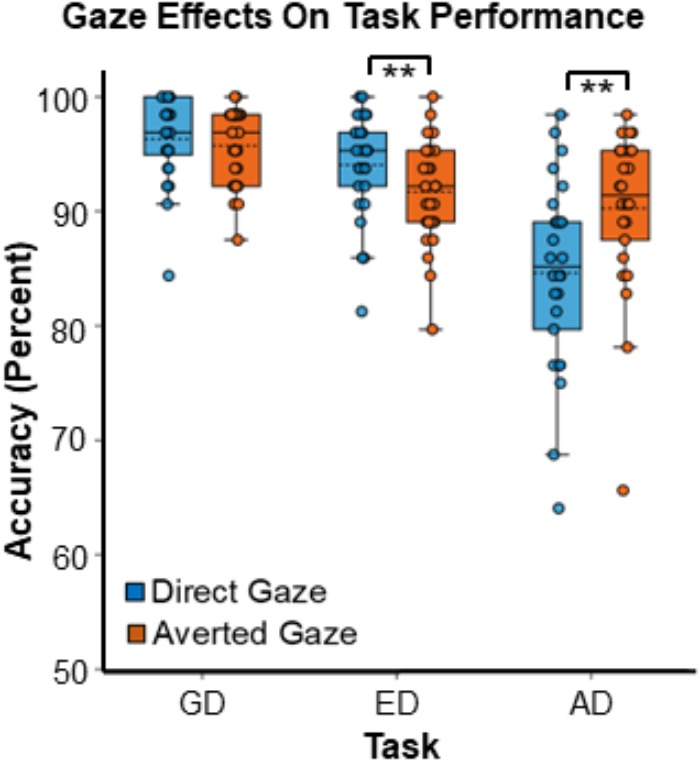
Gaze effects on task accuracy during the three tasks. Data points represent the accuracy for individual participants. Boxes encompass data points between the 25th and 75th percentiles, and within each box the mean (dotted horizontal line) and median (solid horizontal line) are indicated. ^∗∗^Indicates *p* < 0.016, which meets the threshold for significance with Bonferroni correction.

There was a main effect of task on response accuracy,^6^ *F*(2,50) = 31.98, *MSE* = 30.16, *p* < 0.001, $\eta_{p}^{2}$ = 0.56 (Figure 3), driven by greater accuracy in the GD than both the ED task [*t*(25) = 3.71, *SE* = 0.83, *p* = 0.001] and the AD task [*t*(25) = 7.61, *SE* = 1.12, *p* < 0.001], and by greater accuracy in the ED task than in the AD task [*t*(25) = 4.37, *SE* = 1.24, *p* < 0.001].

Although there was no main effect of gaze, *F*(1,25) = 2.82, *MSE* = 12.78, *p* = 0.11, $\eta_{p}^{2}$ = 0.11, there was a strong interaction between gaze direction and task, *F*(1.37, 34.16) = 12.10, *MSE* = 18.70, *p* < 0.001, $\eta_{p}^{2}$ = 0.33 (Figure 3). Planned paired comparisons comparing gaze conditions for each task revealed that participants were more accurate during the AD task in the averted gaze condition than in the direct gaze condition [*t*(25) = 3.18, *SE* = 1.77, *p* = 0.004]. In contrast, during the ED task, participants were more accurate in the direct gaze condition than in the averted gaze condition [*t*(25) = -3.51, *SE* = 0.67, *p* = 0.002]. Finally, there was no accuracy difference between the two gaze conditions for the GD task [*t*(25) = -0.81, *SE* = 0.52, *p* = 0.42]. The accuracy graph was created with BioVinci version 1.1.15 developed by BioTuring Inc.

### EEG Results

#### N170 Analyses

The N170 ANOVA over posterior sites (P9, P10, PO9. PO10, P7, P8) from 130 to 200 ms did not reveal any significant effects of gaze direction, task, nor an interaction between the two.

#### Frontal and Parieto-Occipital Analyses

**FIGURE 4:**
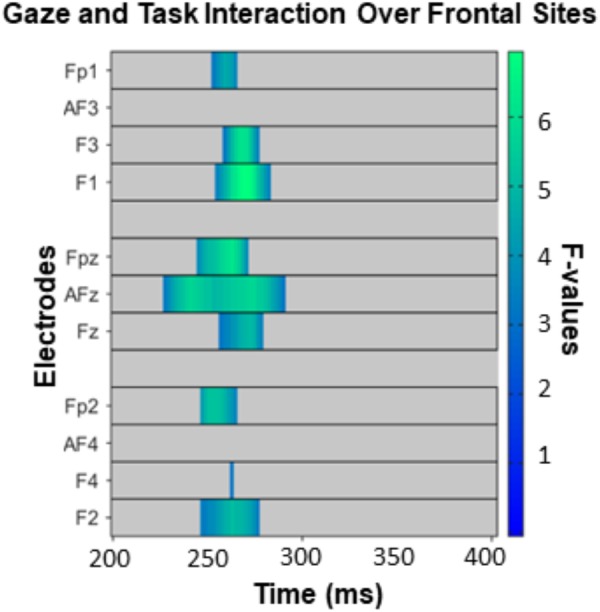
The interaction between task and gaze over frontal sites between 200 and 400 ms, corrected for multiple comparisons with the Permutation Based Cluster Mass technique at *p* < 0.05. Each electrode included in the analysis is plotted on the *y*-axis, while the *x*-axis represents time (post face onset). Colored “blocks” represent significant *F*-values, with the magnitude of the *F*-value plotted according to the right-hand color bar.

**FIGURE 5:**
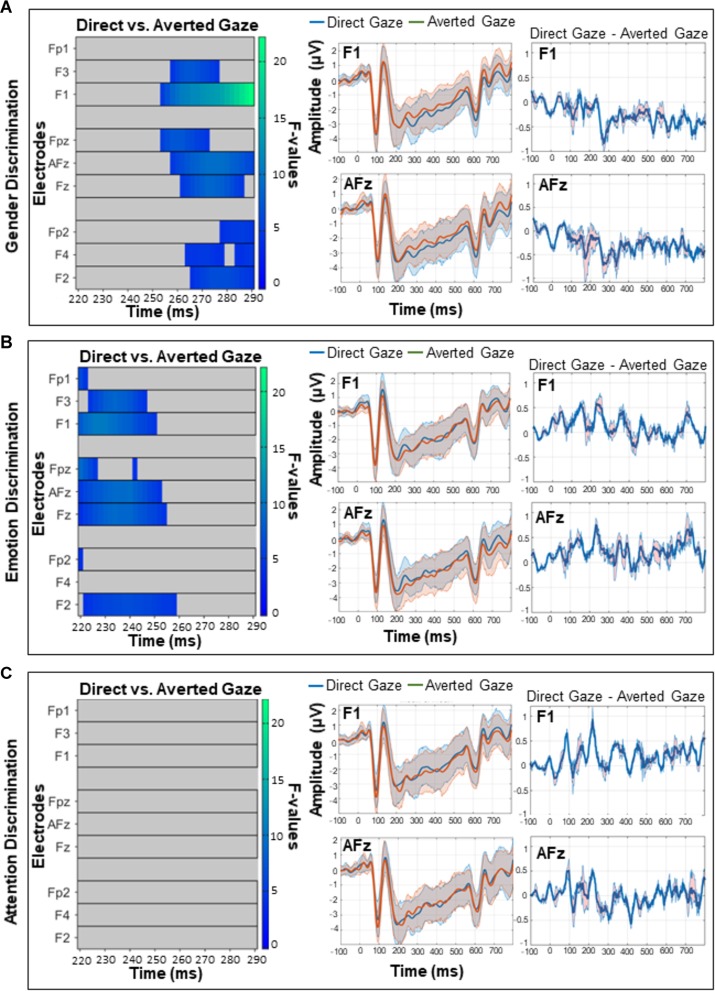
A comparison of direct and averted gaze over frontal sites in the **(A)** gender discrimination **(B)** emotion discrimination, and **(C)** attention discrimination tasks. These *post hoc* analyses were run on the 220–290 ms time widow during which the main omnibus ANOVA yielded a significant interaction (see Figure 4). Left panels depict significant *F*-values corrected with a Permutation Based Cluster Mass technique at *p* < 0.016 (to account for the fact that three follow-up tests were run). Each electrode is plotted on the *y*-axis and each time point (post-face onset) is plotted along the *x*-axis. The color of the “blocks” in these left panels corresponds to the magnitude and direction of significance as indicated by the right-hand color bar. Middle panels depict mean ERP amplitudes and 95% confidence intervals for direct and averted gaze on electrodes F1 and AFz over which the interactions were maximum. Right panels depict the difference between the two gaze conditions (direct gaze amplitude – averted gaze amplitude) on F1 and AFz, with 95% confidence intervals.

The omnibus ANOVA over frontal sites from 200 to 400 ms revealed an interaction between gaze direction and task on ERP amplitudes (Figure 4), but no main effect of gaze or task. While caution must be taken when making inferences about effect latency or location with cluster-based permutation tests (Sassenhagen and Draschkow, 2019), in this latency range the interaction was most pronounced from approximately 220–290 ms over electrodes F3, F1, AFz, and FPz. Our follow-up comparisons during that time window (with *p* < 0.016) of how direct and averted gaze are processed in each task revealed that in the GD task, there were more positive ERP amplitudes for averted gaze than direct gaze (Figure 5A, left). This was most pronounced over F1 and AFz (Figure 5A, middle and right). In contrast, the opposite pattern was seen in the ED task (Figure 5B, left) with direct gaze producing more positive ERP amplitudes than averted gaze (Figure 5B, middle and right). Finally, there was no detectable effect of gaze direction in the AD task (Figure 5C, left, middle and right).

There were no significant effects following the ANOVA over parieto-occipital sites (Pz, POz, PO4, PO3, P1, P2, Oz, O1, O2) from 200 to 500 ms.

#### Exploratory Analysis

**FIGURE 6:**
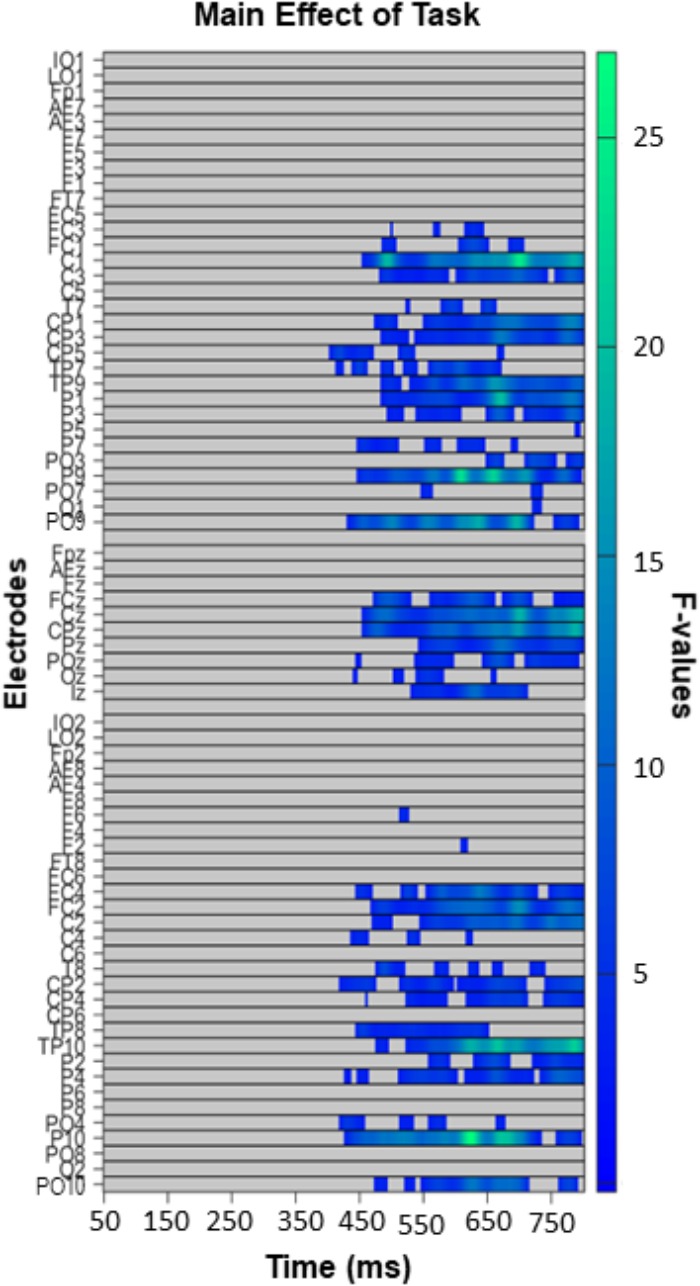
Task effect in the exploratory analysis (50–800 ms, all electrodes), with left panels depicting significant *F*-values corrected with a Permutation Based Cluster Mass technique at *p* < 0.05. Electrodes are plotted on the *y*-axis and time points following face presentation are plotted along the *x*-axis. Colored “blocks” represent significant *F*-values, with the magnitude of the *F*-value plotted according to the right-hand color bar.

**FIGURE 7:**
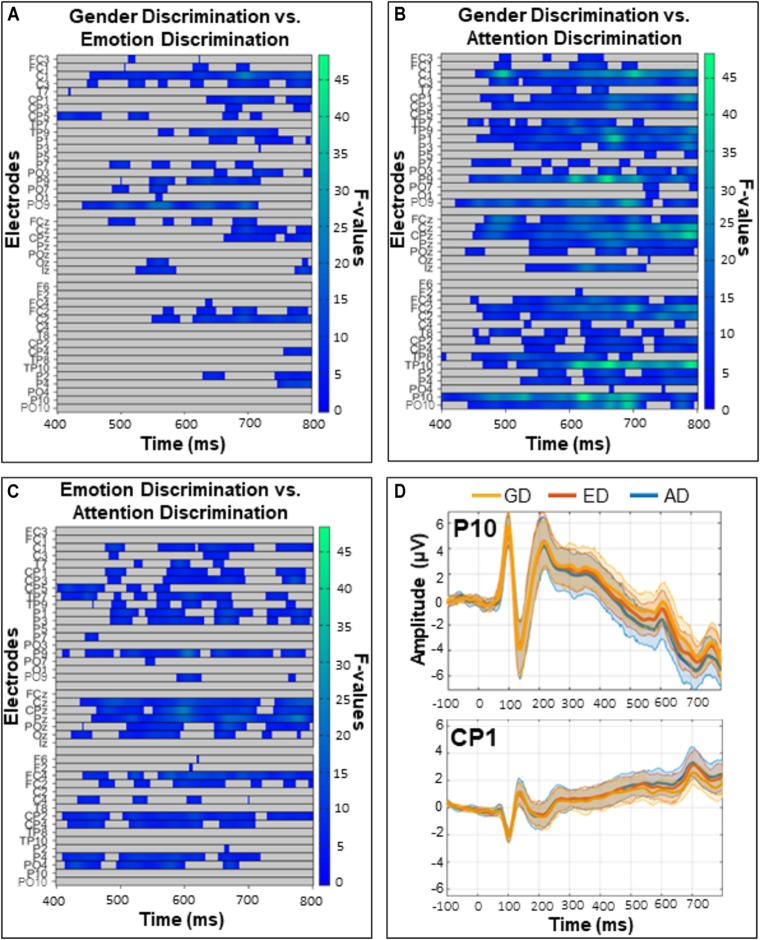
Comparisons of the **(A)** gender and emotion discrimination tasks **(B)** gender and attention discrimination tasks, and **(C)** emotion and attention discrimination tasks. These *post hoc* analyses were run on the 400–800 ms time window during which there was a significant task effect in the omnibus ANOVA (see Figure 5). Left panels depict significant *F*-values corrected with a Permutation Based Cluster Mass technique at *p* < 0.016 (to account for the three follow-up tests). Electrodes are plotted on the *y*-axis and time after face onset is plotted on the *x*-axis. Colored “blocks” represent significant *F*-values, with the magnitude of the *F*-value plotted according to the right-hand color bar. **(D)** Mean ERP amplitudes from representative posterior (P10) and central (CP1) electrodes with 95% confidence intervals.

The exploratory analysis over all electrodes and time-points (excluding the first 50 ms post-face, so between 50 and 800 ms) revealed a widespread main effect of task (Figure 6). It was most pronounced from 400 to 800 ms over posterior and fronto-central sites. Follow up comparisons indicated that this effect was driven by differences between the GD and ED tasks (Figure 7A), the GD and AD tasks (Figure 7B), and the ED and AD tasks (Figure 7C). Over posterior sites, ERP amplitudes were most negative in the AD task, intermediate in the ED task, and most positive in the GD task (Figure 7D, top). The opposite pattern was found over fronto-central sites (Figure 7D, bottom).

## Discussion

The importance of eye-gaze processing during social interactions is undisputed (Kleinke, 1986; Emery, 2000; George and Conty, 2008; Itier and Batty, 2009 for reviews) and is particularly evident in disorders which feature both eye-gaze avoidance and social impairment, including Autism Spectrum Disorder (Pelphrey et al., 2002; Senju and Johnson, 2009; Madipakkam et al., 2017) and Social Anxiety Disorder (Schneier et al., 2011). The clinical significance of altered eye-gaze processing has led to a field of research devoted to understanding how direct and averted gaze are processed in the brain, and how we use them as cues to inform our social interactions.

While there has been much interest in examining the neural correlates of eye-gaze processing, there does not seem to be a consensus about where and when direct and averted gaze are differentiated in the brain. One of the likely reasons for this lack of consensus is that the experimental tasks in studies of gaze processing vary quite substantially (Hoffman and Haxby, 2000; Hooker et al., 2003; Carrick et al., 2007; Latinus et al., 2015; Burra et al., 2018). Given that direct and averted gaze can be interpreted differently in different social circumstances (Hamilton, 2016), it is likely that these gaze cues are processed differently depending on the type of task participants are asked to complete. To this end, we examined how viewing individuals with direct and averted gaze would affect performance during three different tasks commonly used in the field, in a within-subjects design. Those tasks have been previously used to study gaze processing in separate samples (one task at a time) and included an Emotion Discrimination (ED) task, where participants discriminated between two facial expressions, an Attention Discrimination (AD) task that required participants to infer the direction of the individual’s attention based on gaze cues and a Gender Discrimination (GD) task. We found that direct and averted gaze elicited different behavioral effects depending on the task that participants were performing (Figure 3). Direct gaze was associated with better accuracy than averted gaze during the ED task, while averted gaze was associated with better accuracy in the AD task. However, there was no significant effect of gaze direction on performance in the GD task.

Although we believe our behavioral interactions between gaze and task reflect interactions between gaze processing and AD and ED task demands, other potential explanations should be noted. First, previous literature has reported that direct gaze has a facilitatory effect on a myriad of tasks including capturing attention (Yokoyama et al., 2014), facilitating recognition memory (Vuilleumier et al., 2005), and gender discrimination (Macrae et al., 2002; Burra et al., 2018; but see Vuilleumier et al., 2005). While it is possible that a general facilitatory effect of direct gaze may explain our behavioral findings in the ED task, we do not believe this is the case because no significant effect of gaze direction was found in the GD task. This would suggest that the facilitatory effect of direct gaze during the ED task was above any standard facilitation effect. Furthermore, the AD task was associated with worse performance for direct gaze, which goes against this explanation. It is important to highlight that all previous studies reporting facilitated effects for direct gaze studied only one task at a time, in contrast to the present within-subject design which directly compared three tasks in the same individuals.

A similar argument could be made regarding the possibility of gaze cuing effects influencing the results. Given that the gaze cuing literature suggests that spontaneous attention shifts occur toward gazed-at locations even when gaze direction is task irrelevant (Friesen and Kingstone, 1998; Driver et al., 1999), one could argue that averted gaze may have oriented participants’ attention away from the stimuli during the tasks. However, there is no reason why this potential attention shift should have affected tasks differently, and because there was no effect of gaze direction on accuracy in the GD task, and opposite effects of gaze direction in the ED and AD tasks, it is unlikely that covert attention shifts in the direction of averted gaze could explain the pattern of results.

It must be noted that others have reported that direct gaze is associated with improved ED. Adams and Kleck (2003, 2005) and Sander et al. (2007) also found that angry and happy facial expressions (as used in the present study) were perceived more easily when paired with direct gaze than with averted gaze. However, they also found that fear and sadness were perceived more easily when paired with *averted* gaze than with direct gaze. Adams and Kleck (2003) proposed that direct gaze enhances the perception of facial expressions signaling behavioral approach from the gazer (e.g., angry and happy expressions), while averted gaze enhances the perception of facial expressions signaling behavioral avoidance (e.g., sadness and fear) due to a “shared signal” between gaze and emotion expression decoding. Although the support for the shared signal hypothesis was largely found to be tied to the specific stimuli used (Graham and LaBar, 2007; Bindemann et al., 2008), it is still possible that gaze direction may facilitate or impair ED differently depending on the emotional expression on the face. Replication of the present findings and extension to more facial expressions is needed to examine this possibility further.

There is also another potential explanation for our behavioral gaze effects, which concerns the inherently self-referential nature of direct gaze (Conty et al., 2016). Direct gaze signals to us that we are the direction of someone’s attention (George and Conty, 2008; Itier and Batty, 2009; Conty et al., 2016), and has been shown to produce similar fMRI brain activation as hearing one’s name being called (Kampe et al., 2003). Gaze processing has also been shown to interact with the self-relevance of contextual sentences at the ERP level (McCrackin and Itier, 2018c). In the attention discrimination task, participants indicated whether the individuals were directing their attention at them or away from them. This may have primed self-referential processing, which could have impacted how direct gaze was processed. However, if this was the case, one would expect participants to be more accurate at responding to direct gaze faces in the AD task, while the opposite was observed. In fact, if anything, the pattern of results (Figure 3) suggests that direct gaze hindered performance in the AD task (as opposed to a true accuracy benefit for the averted gaze condition).

We also found that gaze processing interacted with task at the ERP level, although the pattern of results did not map directly onto the pattern of behavioral results. Gaze processing differed between the three tasks from 200 to 400 ms over frontal sites. While there was no gaze difference in ERP amplitudes in the AD task over these sites, direct gaze elicited more positive amplitudes than averted gaze in the ED task, but less positive amplitudes than averted gaze in the GD task. The interaction between gaze direction and task indicated that these two effects overlapped in time, although the ED gaze effect appeared earlier (around 220 ms) than the GD gaze effect (around 255 ms). Interestingly, the ED gaze activity occurs in a time-window during which decoding of emotions typically occurs. The Early Posterior Negativity EPN – that typically differentiates between different facial expressions, in particular fearful and angry compared to happy facial expressions (e.g., Sato et al., 2001; Schupp et al., 2006; Herbert et al., 2008; Kissler et al., 2009; Wronka and Walentowska, 2011; Rellecke et al., 2012; Neath and Itier, 2015; Neath-Tavares and Itier, 2016), is often reported between 150 and 250 ms and up to 350 ms at posterior sites. Given that direct gaze has been implicated in emotion processing (Kampe et al., 2001; Strick et al., 2008; Hamilton, 2016) and affects participants’ arousal (Nichols and Champness, 1971; Conty et al., 2010; McCrackin and Itier, 2018c) and introspective reporting of emotional state (Baltazar et al., 2014), the present frontal activation in the ED task may be indicative of overlap between the neural correlates associated with emotion processing and gaze processing.

Despite its excellent temporal resolution, EEG has poor spatial resolution, so caution must be taken when making inferences about possible neural generators. Nevertheless, we speculate that the frontal activity recorded is linked to orbitofrontal (OFC) activity, given the involvement of the OFC in emotion processing, gaze processing and higher order theory of mind tasks (Calder et al., 2002; Amodio and Frith, 2006; Conty et al., 2007; Dixon et al., 2017). The 220–290 ms during which the task by gaze interaction was found significant at this frontal cluster falls in between timings reported by two independent studies to be sensitive to gaze (Conty et al., 2007) and task (Sabbagh et al., 2004), respectively. Conty et al. (2007) reported OFC activation to differentiate between direct and averted gaze from 190 to 220 ms (picked up first over frontocentral and centroparietal sites, e.g., Fz, Cz, then later over occipital-temporal sites, e.g., P9, P10). In another study, source localization pointed to the OFC as the source of ERP amplitude differences found between 270 and 400 ms and differentiating between a GD task and an ED task close to our own (over frontal sites including FP2 and F4, as well as parieto-occipital sites), which asked participants to decode emotional state from eye-regions with direct gaze (Sabbagh et al., 2004). We thus find it plausible that the OFC would be involved in the gaze by task interaction picked up at frontal sites during similar timing.

One of the limitations of this study concerns the differences between the demands associated with each task, and it is unclear what differences between tasks are responsible for the differences in how gaze was processed during each. For example, while we assume that the key factor differentiating the ED from the GD and AD tasks is the recruitment of frontocentral emotion processing centers in the ED task, in particular the orbitofrontal cortex, the tasks also differ in terms of featural versus holistic processing. Indeed, the AD task may have required featural processing of the eyes, while both ED and GD judgments are generally considered to require holistic face processing (e.g., McKelvie, 1995; Prkachin, 2003; Calder and Jansen, 2005; Zhao and Hayward, 2010). However, as opposite gaze effects were seen between the GD and ED tasks at the neural level, this featural versus holistic processing difference cannot easily explain our neural interaction.

In contrast, a featural/holistic difference in processing may account for overall task differences found from 400 and 800 ms post-stimulus that may be related to task difficulty. Over occipitotemporal sites, the most positive ERP amplitudes were elicited by the GD task, intermediate amplitudes by the ED task, and the most negative amplitudes were elicited by the AD task. The reverse pattern was seen over centro-parietal sites, likely reflecting the opposite end of the same dipole. Similar task effects have been reported in studies in which participants used eye-regions (Sabbagh et al., 2004) or faces (Itier and Neath-Tavares, 2017 but see Rellecke et al., 2012 for null results) to complete similar ED and GD tasks. Itier and Neath-Tavares (2017) reported more positive ERPs in the GD than the ED task over posterior sites but at much earlier timings (from 200 to 350 ms, the latest measured time window due to much shorter response times). Sabbagh et al. (2004) reported more positive ERPs for the ED task than the GD task over posterior, central and parietal sites at a timing closer to our own timing (300–500 ms, where as our task effect began at 400 ms). These timing differences may be related to the fact that in the present study and the Sabbagh (2004) study, participants were asked to wait until the response prompt to press the keys while in the Itier and Neath-Tavares (2017) study, responses occurred as soon as possible after the presentation of the stimulus. Similar task effects have also been found when participants were asked to perform visual discrimination tasks with differing levels of complexity (Senkowski and Herrmann, 2002). Our behavioral data support the idea that task complexity might be responsible for these general effects of tasks, given the accuracy gradient followed the same pattern as the ERP amplitude gradient. Accuracy was indeed highest in the GD task, intermediate in the ED task, and worst in the AD task. Similar response time (Wronka and Walentowska, 2011; Rellecke et al., 2012) and accuracy (Wronka and Walentowska, 2011) gradients were previously reported by groups using similar GD and ED tasks. Overall, the general task effects seen at the ERP level seem related to task difficulty and future studies could investigate whether this difficulty is related to featural/holistic processing differences or to other task-specific factors.

We should also note that it was surprising to find neither a main effect of gaze direction, nor an interaction between gaze and task, over posterior sites during the 130–200 ms window encompassing the N1710, given past reports of gaze effects on this ERP component (Puce et al., 2000; Watanabe et al., 2002, 2006; Conty et al., 2007; Itier et al., 2007; George and Conty, 2008; Itier and Batty, 2009; Pönkänen et al., 2010; Latinus et al., 2015; Rossi et al., 2015; Burra et al., 2017). These previous reports have been quite mixed, with some finding enhanced N170 amplitudes in response to averted gaze (Puce et al., 2000; Watanabe et al., 2002; Itier et al., 2007; Latinus et al., 2015; Rossi et al., 2015), some to direct gaze (Watanabe et al., 2006; Conty et al., 2007; Pönkänen et al., 2010; Burra et al., 2017), and others, like the present study, finding no gaze effect at all (Taylor et al., 2001; Schweinberger et al., 2007; Brefczynski-Lewis et al., 2011). One possibility is that there is a lot of variation in how gaze is processed at the individual level over these sites (the N170 itself can range in latency from 130 to 200 ms between individuals). While there may be some similarities in timing and location, significant individual differences could have impacted our ability to detect gaze effects at the group level using a mass-univariate approach. Moreover, this literature on gaze effect almost always used neutral faces, while the present study used emotional expressions, which may have impacted the early processing of gaze. The other alternative is that previously reported findings regarding N170 modulations by gaze were type I errors that may be related to the lack of control of gaze position. Indeed, as far as we know, the present study is the first ERP study on gaze perception to have controlled for gaze position using a gaze-contingent approach, a particularly important aspect given the growing literature showing modulations of the N170 amplitude with gaze fixation location, in particular to the eyes (de Lissa et al., 2014; Nemrodov et al., 2014; Neath and Itier, 2015; Neath-Tavares and Itier, 2016; Itier and Preston, 2018; Parkington and Itier, 2018). Those possible caveats represent an important topic for further research to address. In any case, from the present (and unique) within-subject design, there is no evidence of early gaze effects during the time window encompassing the N170 component, as least when using facial expressions of emotion.

In summary, the present study is one of the first ERP investigations demonstrating that direct and averted gaze are processed differently during emotion, attention and gender discrimination judgments performed by the same participants. Gaze direction did not affect GD task performance, while processing direct gaze facilitated emotion discrimination relative to averted gaze, and processing averted gaze facilitated the attention direction judgment relative to direct gaze. These results provide support for the idea that gaze perception impacts attention and emotion discrimination judgments, which are likely key initial steps in our everyday theory of mind. If perceiving direct gaze facilitates ED and perceiving averted gaze facilitates AD, avoiding the eye-region will prevent this facilitation from occurring. Accordingly, our findings are in line with the assumption that the eye-gaze avoidance characteristic of autism spectrum disorder (e.g., Pelphrey et al., 2002; Senju and Johnson, 2009) may be contributing to impairments in emotion discrimination (Humphreys et al., 2007; Clark et al., 2008) and joint attention (Bruinsma et al., 2004), and perhaps even to the theory of mind impairments found in this condition (Baron-Cohen, 1995; Senju et al., 2009). Furthermore, our ERP findings provide a potential mechanism to explain how this may occur in ED: avoiding the eyes may result in less recruitment of frontal areas that process both gaze and emotion. If so, behavioral therapies encouraging exploration of the eye-region may have the added benefit of improving emotion discrimination and potentially theory of mind.

## Ethics Statement

This study was carried out in accordance with the recommendations of the UW Research Ethics Board, with written informed consent from all subjects. All participants gave written informed consent in accordance with the Declaration of Helsinki. The protocol was approved by the UW Research Ethics Board.

## Author Contributions

SM and RI involved in the early conceptualization and experimental design for this project and revised the draft numerous times together. SM programmed the experiments, created the stimuli, ran the participants, processed the data, analyzed the data with advice from RI, and wrote the initial draft of the manuscript.

## Conflict of Interest Statement

The authors declare that the research was conducted in the absence of any commercial or financial relationships that could be construed as a potential conflict of interest.

## Footnotes

1 Identities 10, 15, 19, 30, 31, 32, 33, 37 were used in the study blocks, while identities 7 and 14 were used in the practice block.

2 The Biosemi Active-Two system does not use an actual recording reference site.

3 http://erpinfo.org/erplab

4 Trials per condition: Direct GD = 99.46 (*SD* = 22.49), Averted GD = 98.50 (*SD* = 23.91), Direct ED = 98.07, *SD* = 24.00, Averted ED = 96.69 (*SD* = 28.25), Direct AD = 94.27 (*SD* = 21.03), Averted AD = 96.69 (*SD* = 28.25).

5 https://osf.io/am4zv/?view_only=eac91ae8a07e44f7ab5aca550fc19da2

6 For the interested reader, the RT time-locked to the onset of the answer prompt displayed a similar pattern as the accuracy data: the main effect of task [*F*(1.40,34.96) = 13.14, *MSE* = 13908.60, *p* < 0.001, $\eta_{p}^{2}$ = 0.34] was driven by faster responses during the GD than both the ED [*t*(25) = -4.24, *SE* = 11.52, *p* < 0.001] and AD [*t*(25) = -4.29, *SE* = 23.09, *p* < 0.001] tasks, as well as faster responses during the ED than the AD task [*t*(25) = -2.35, *SE* = 21.36, *p* = 0.027]. There was no main effect of gaze (*F* = 1.02, *p* = 0.32), though there was a significant interaction between task and gaze [*F*(2,50) = 6.17, *MSE* = 3568.78, *p* = 0.004, $\eta_{p}^{2}$ = 0.20]. RTs were faster for the averted gaze than the direct gaze condition during the AD task [*t*(25) = -2.72, *SE* = 17.96, *p* = 0.012]. The opposite pattern, thought not significant, was observed in the ED task, with faster RTs during the direct gaze than the averted gaze condition [*t*(25) = 1.80, *SE* = 14.57, *p* = 0.084]. There was no RT difference between gaze conditions for the GD task [*t*(25) = 0.024, *SE* = 9.94, *p* = 0.98].
